# Antioxidant Protection of NADPH-Depleted Oligodendrocyte Precursor Cells Is Dependent on Supply of Reduced Glutathione

**DOI:** 10.1177/1759091416660404

**Published:** 2016-07-21

**Authors:** Ewa Kilanczyk, Sujata Saraswat Ohri, Scott R. Whittemore, Michal Hetman

**Affiliations:** 1 Kentucky Spinal Cord Injury Research Center, University of Louisville, KY, USA; 2 Department of Neurological Surgery, University of Louisville, KY, USA; 3 Department of Anatomical Sciences and Neurobiology, University of Louisville, KY, USA; 4 Department of Pharmacology and Toxicology, University of Louisville, KY, USA

**Keywords:** pentose phosphate pathway, metabolism, oxidative stress, white matter, oligodendrocytes, demyelination

## Abstract

The pentose phosphate pathway is the main source of NADPH, which by reducing oxidized glutathione, contributes to antioxidant defenses. Although oxidative stress plays a major role in white matter injury, significance of NADPH for oligodendrocyte survival has not been yet investigated. It is reported here that the NADPH antimetabolite 6-amino-NADP (6AN) was cytotoxic to cultured adult rat spinal cord oligodendrocyte precursor cells (OPCs) as well as OPC-derived oligodendrocytes. The 6AN-induced necrosis was preceded by increased production of superoxide, NADPH depletion, and lower supply of reduced glutathione. Moreover, survival of NADPH-depleted OPCs was improved by the antioxidant drug trolox. Such cells were also protected by physiological concentrations of the neurosteroid dehydroepiandrosterone (10^−8^ M). The protection by dehydroepiandrosterone was associated with restoration of reduced glutathione, but not NADPH, and was sensitive to inhibition of glutathione synthesis. A similar protective mechanism was engaged by the cAMP activator forskolin or the G protein-coupled estrogen receptor (GPER/GPR30) ligand G1. Finally, treatment with the glutathione precursor N-acetyl cysteine reduced cytotoxicity of 6AN. Taken together, NADPH is critical for survival of OPCs by supporting their antioxidant defenses. Consequently, injury-associated inhibition of the pentose phosphate pathway may be detrimental for the myelination or remyelination potential of the white matter. Conversely, steroid hormones and cAMP activators may promote survival of NADPH-deprived OPCs by increasing a NADPH-independent supply of reduced glutathione. Therefore, maintenance of glutathione homeostasis appears as a critical effector mechanism for OPC protection against NADPH depletion and preservation of the regenerative potential of the injured white matter.

## Introduction

Oxidative stress is a critical mediator of oligodendrocyte death and results in white matter damage that occurs after traumatic spinal cord injury (SCI) or in multiple sclerosis (McTigue and Tripathi, 2008). It has been proposed that high susceptibility of oligodendrocytes to oxidative damage is due to the limited levels of reduced glutathione which is the key antioxidant molecule of the cell (McTigue and Tripathi, 2008). Moreover, at least during myelination, intense membrane synthesis requires these cells to maintain high rates of lipid and protein production further adding to oxidative stress susceptibility (McTigue and Tripathi, 2008). Finally, large iron stores of oligodendrocytes provide another layer of oxidative hypersensitivity by promoting generation of highly toxic reactive oxygen species (ROS; McTigue and Tripathi, 2008). Therefore, regulators and effectors of oligodendrocyte antioxidant defenses may provide therapeutic targets for white matter protection.

NADPH is a critical component of antioxidant defenses as it is required for reduction of oxidized glutathione and may also work directly as an antioxidant (Stanton, 2012). The major cellular source of NADPH is glucose oxidation through the pentose phosphate pathway (PPP; Stanton, 2012). The PPP generates NADPH by its rate-limiting enzyme, the 6-phosphate dehydrogenase (G6PDH) and also by the 6-phospho gluconate dehydrogenase. Indeed, the PPP is critical for antioxidant defenses in various cell types including neurons (Pandolfi et al., 1995; Herrero-Mendez et al., 2009; Stanton, 2012).

Cultured OPCs have highly active PPP (Sanchez-Abarca et al., 2001). In addition, as compared with cultured neurons or astrocytes, their glucose consumption is higher with a substantial portion of that substrate being metabolized via the PPP (Sanchez-Abarca et al., 2001). Mature oligodendrocytes in culture maintain highly active PPP, but its activity is lower than in OPCs and comparable to astrocytes (Amaral et al., 2016). These cell culture findings are likely relevant to the intact nervous system, as oligodendrocytes are known for their very high expression or activity of various PPP enzymes including G6PDH or transaldolase (Banki et al., 1994; Kugler, 1994). In oligodendrocytes, such a metabolic bias correlates with high activity of lipid synthesis (McTigue and Tripathi, 2008). Indeed, NADPH and glucose-derived acetyl-CoA are necessary substrates to produce lipids. In addition, oligodendrocytes may be highly dependent on NADPH and the PPP for their resistance to oxidative damage. However, antioxidant role of NADPH has never been directly demonstrated in these cells.

The NADPH antimetabolite 6-amino-NADP (6AN) blocks various NADP/NADPH-dependent enzymes including G6PDH and 6-phospho gluconate dehydrogenase (Kohler et al., 1970). Administration of 6AN to rats disrupted the spinal cord PPP, induced paralysis, and triggered extensive myelin damage, including loss of oligodendrocytes (Miyoshi et al., 1978; Cheeseman and Hothersall, 1988). While these findings suggest a critical role of NADPH supply for survival of oligodendrocytes, cell autonomous nature of these effects has not been demonstrated.

NADPH supply can be also compromised by inhibiting G6PDH with at least 10^−4^ M concentrations of the endogenous steroid dehydroepiandrosterone (DHEA; Shantz et al., 1989). However, improved locomotor outcomes and white matter sparing were reported when DHEA was applied to mice with a moderate thoracic contusive SCI (Fiore et al., 2004). Such effects may be related to various receptor-mediated activities of DHEA that include modulation of neurodevelopment and neuroprotection (Charalampopoulos et al., 2008; Maninger et al., 2009). Conversely, as NADPH oxidase contributes to oxidative stress and oligodendrocyte death in injured spinal cord, reducing NADPH supply could also be protective (Johnstone et al., 2013). Indeed, by depleting NADPH, 6AN reduces NADPH oxidase activation and attenuates heart tissue injury following experimental ischemia or reperfusion (Zuurbier et al., 2004). Hence, role of NADPH in oligodendrocyte survival is not entirely clear and has never been tested at the cellular level. This study was initiated to evaluate NADPH contribution to survival of cultured primary oligodendrocyte precursor cells (OPCs) from adult rat spinal cord.

## Materials and Methods

### Reagents

Unless stated otherwise, all reagents were obtained from Sigma, VWR, or Fisher Scientific.

### Cell Culture

Adult rat spinal cord OPCs were isolated from Fisher rats by immunopanning with an O4 antibody as described earlier (Cheng et al., 2007). OPCs were cultured on poly-l-lysine/laminin-coated plastic dishes in DMEM-F12 (Invitrogen) medium that was supplemented with 2.5 g/l NaHCO_3_, 1% N_2_ supplement, 2% B27 supplement, 1% penicillin or streptomycin, 0.01% BSA, 5 µg/ml insulin, 40 ng/ml FGF2 (Millipore), and 20 ng/ml PDGFaa, and approximately 15,000 cells/cm^2^ were seeded on a PDL/laminin-coated 10 cm tissue culture plate. To induce oligodendrocytic differentiation, OPCs were cultured for 4 days in mitogen-free media supplemented with 30 ng/ml triiodothyronine (T3) as described earlier (Tokumoto et al., 1999). Schwann cells were isolated from adult mice sciatic nerves and cultured following standard procedures (Morrissey et al., 1991). Experiments were performed until 18th or 10th passages for OPCs and Schwann cells, respectively. Such OPCs stained positive for OPC markers (NG2 and CNPase) and retained the potential to produce myelin basic protein (MBP)-positive oligodendrocytes after differentiation (Cheng et al., 2007 and data not shown) .

### Cell Treatments

Cells were seeded on 96-well plates (10,000 cells per well). After 24 h, cells were cotreated with different drugs and incubated for next 24 h. For cell culture studies, 6AN (6-aminonicotinamide), trolox ([±]-6-hydroxy-2,5,7,8-tetramethylchromane-2-carboxylic acid), H89 dihydrochloride hydrate (N-[2-(p-Bromocinnamylamino)ethyl]-5-isoquinolinesulfonamide dihydrochloride), forskolin, buthionine sulfoximine (BSO), and N-acetyl cysteine were dissolved in water; G1, DHEA, etoposide, and tunicamycin were dissolved in dimethyl sulfoxide (DMSO). The final concentration of DMSO in the cell culture medium was less than 0.5%.

Phase contrast imaging was done using a 10× lens and the Zeiss AxioCam Inverted microscope system with AxioVision software.

### Lactate Dehydrogenase Release

Samples of culture media were collected from OPCs that were cultured in 96-well plates and challenged with 6AN. Lactate dehydrogenase (LDH) release was determined using Pierce™ LDH Cytotoxicity Assay Kit (Life Technologies) following manufacturer's recommendations.

### MTT Assay

For MTT (3-(4, 5-dimethylthiazol-2-yl)-2,5-diphenyltetrazolium bromide) assays, OPCs were cultured in 96-well plates. Standard methodology was applied as described earlier (Hetman et al., 1999).

### NADPH/NADP^+^ Levels

NADP^+^ and NADPH levels were determined using a previously described method (Zhang et al., 2000). Briefly, OPCs' extracts were prepared by resuspending cells in 0.1 M Tris-HCl (pH 8.0), 0.01 M EDTA, 0.05% Triton X-100 followed by sonication for 2 min with 30 s interval and centrifugation at 2,500 × g, 4℃ for 5 min. The supernatant was collected and analyzed for NADP + and NADPH in three steps as follows: (a) a spectrophotometric reading was taken using untreated extract to determine total amount of NADPH and NADH in the sample (absorbance value A1 at 340 nm), (b) an aliquot of the extract was incubated with G6PDH (Sigma) to convert all of the NADP + in the sample to NADPH, and its levels were determined as in step 1 to produce absorbance value A2, (c) another aliquot of the extract was incubated with glutathione reductase (Calbiochem) to convert all of NADPH to NADP^+^, and its levels were determined as in Steps 1 and 2 to obtain absorbance value A3. A1 to A3 and A1 to A2 represented the total amount of NADPH and NADP^+^, respectively. Results were expressed as µmol of NADPH/NADP^+^ per milligram of protein.

### Glutathione Assay

OPCs were lysed in 1% sulfosalicylic acid, centrifuged at 13,000 × g for 5 min at 4℃, and supernatants were collected. Contents of both reduced- (GSH) and oxidized glutathione (GSSG) were determined as described earlier (Garcia-Nogales et al., 1999). Briefly, to determine GSH + GSSG content (GSx), supernatants were treated with glutathione reductase and absorbance was monitored at 405 nm. GSSG was measured in a similar manner after GSH was derivatized using 2-vinylpiridine. Data were extrapolated to those obtained with GSSG standards (0–5 μM for GSSG; 0–50 μM for GSx). Results were expressed as µmol of GSH/GSSG per milligram of protein.

### NBT Assay to Measure Superoxide

A published procedure was followed with modifications (Choi et al., 2006). Rat OPCs, which were grown in 96-well plates, were treated with 6AN or DHEA followed by medium replacement with fresh medium that was supplemented with 1 mg/ml nitro blue tetrazolium chloride (NBT). After 1 h incubation (37℃, 5% CO_2_), cells were washed twice with warm PBS followed by the addition of 50 μl 2 M KOH to solubilize cell membranes and then 50 μl of DMSO to dissolve blue formazan with gentle shaking for 10 min at room temperature. The dissolved NBT solution was then transferred to a 96-well plate, and absorbance was read at 620 nm using a microplate reader.

### Mitochondrial Potential Assay

A cell-permeable fluorescent indicator of mitochondrial potential, tetramethylrhodamine methyl ester (TMRM) was used at 20 nm following a published protocol (Joshi and Bakowska, 2011). After 45 min TMRM loading, rat OPCs were imaged using an epifluorescent microscopy system (Zeiss Observer.Z1; × 40 lens) at 561 nm excitation. Images were taken using AxioVision software at identical exposure times. Fluorescence intensity of TMRM was analyzed using ImageJ (“Area” parameter) at the single cell level.

### Western Blotting

Western blotting using rabbit anticleaved caspase 3 antibody (1:1000; Cell signaling) was performed using standard procedures. To verify equal loading, blots were reprobed using mouse anti-GAPDH antibody (Alexis Biochemicals) at a 1:3000 dilution.

### Protein Assay

Protein concentrations were determined using the Bradford method and the commercially available Bradford reagent (BioRad).

### Statistical Analysis

Data were analyzed using the nonparametric Mann–Whitney test (*U* test) or analysis of variance (followed by post hoc tests) as indicated.

## Results

### The NADPH Antimetabolite 6AN Kills Oligodendrocytes

**Figure 1.**
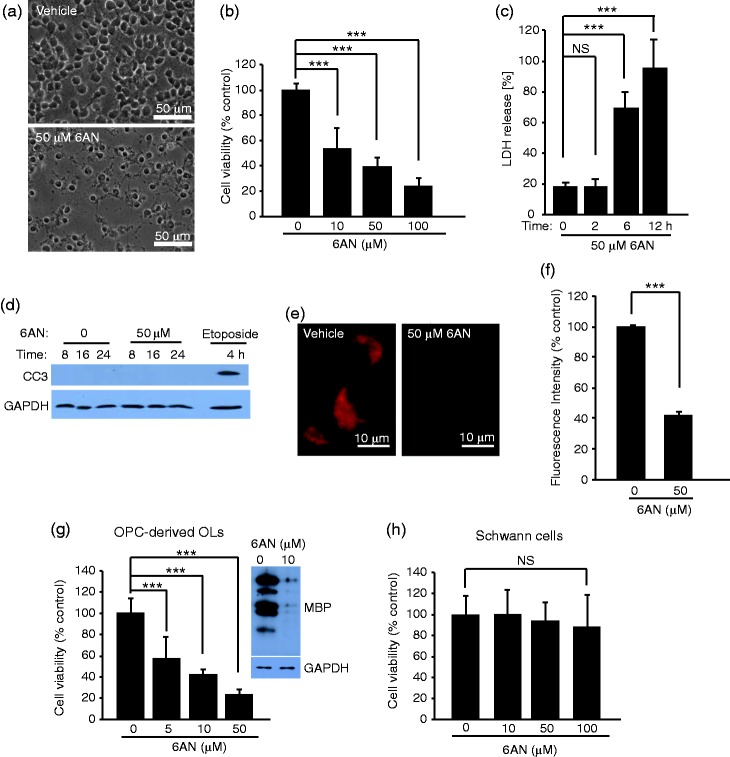
The NADPH antimetabolite 6-amino-NADP (6AN) is toxic to oligodendrocyte precursor cells and oligodendrocyte precursor cell-derived oligodendrocytes. Undifferentiated OPCs (a–f), oligodendrocytes (g), and Schwann cells (h) were treated with 6AN as indicated. (a) Representative phase contrast micrographs depict declining density of OPC cultures that were exposed to 6AN for 24 h. (b) MTT cell survival assays revealed declining number of viable OPCs in response to such treatment. (c) LDH release assay revealed extensive plasma membrane permeabilization as early as 6 h after adding 6AN. Such a response suggests that necrosis is the major cause of reduced viability in 6AN-treated OPCs. (d) Western blot with an antibody specific for the activated form of the apoptotic protease caspase-3 (cleaved caspase-3, CC3) revealed caspase activation in response to the DNA damaging drug etoposide (1 μM) but not 50 μM 6AN. Equal loading was confirmed by reprobing the membrane with an antibody against GAPDH. (e) and (f) Cells that were treated with 6AN for 6 h were loaded with the mitochondrial potential sensor TMRM. (e) In vehicle-treated control cells, red fluorescence of TMRM in the perikaryal region reflects distribution of functional mitochondria as confirmed by no signal in cells that were treated with the mitochondria uncoupling chemical FCCP (1 μM, data not shown). TMRM fluorescence was reduced in 6AN-treated cells. (f) Quantification of TMRM fluorescence intensity revealed a 60% decline following 6AN exposure. After 2 h treatment with 6AN, TMRM fluorescence was similar as in vehicle-treated cells (not shown). (g) Declining viability was also observed in OPC-derived oligodendrocytes that were treated with 6AN for 24 h. In such cultures, Western blot revealed sharply reduced MBP expression suggesting a high sensitivity of maturing oligodendrocytes to PPP inhibition. Equal loading of the blot was confirmed by reprobing of the membrane for GAPDH. (f) Primary mouse Schwann cells were less sensitive to PPP inhibition than OPCs as revealed by the MTT assay at 72 h after initiation of 6AN treatment. These differences are unlikely due to differential sensitivity of mouse versus rat cells as mouse brain OPCs were as sensitive to 6AN as rat spinal cord OPCs (not shown). In (b), (c), (g), (h), data represent averages ± *SD* of nine sister cultures from three independent experiments; in (f), averages ± *SD* of at least 54 cells from three independent experiments are shown; *ns, p* > .05, ***p < .001 (analysis of variance).

In OPC cultures that were treated with 6AN for 24 h., a strong reduction of cell density was observed (Figure 1(a)). Cytotoxic response to 6AN was confirmed by MTT survival assays (Figure 1(b)). Moreover, as soon as 6 h after the onset of 6AN exposure, 70% of all intracellular LDH activity was released into the media suggesting plasma membrane permeabilization (Figure 1(c)). Such a cytotoxic response was not accompanied by activation of the apoptotic effector protease caspase-3 (Figure 1(d)). In contrast, activation of caspase-3 was observed in OPCs that were treated with the DNA damaging drug etoposide (Figure 1(d)). Hence, 6AN-induced death of OPCs appears to be necrotic as indicated by relatively early permeabilaztion of the plasma membrane and lack of caspase-3 activation.

As necrosis is often associated with early damage of mitochondria that leads to disruption of cellular energy supply, function of mitochondria was probed with the fluorescent mitochondrial potential sensor TMRM. In control conditions, strong TMRM signal was present in the perikaryal region of OPCs consistent with intracellular distribution of mitochondria (Figure 1(e)). No change in TMRM fluorescence was observed at 2 h after adding 6AN (not shown). However, after 6 h treatment with 6AN, TMRM signal intensity was down by 60% suggesting mitochondrial damage (Figure 1(e) and (f)). These observations further support a possibility that reducing NADPH supply elicits necrosis of OPCs.

When OPCs were differentiated to oligodendrocytes, they were still sensitive to 6AN as indicated by declining metabolism of MTT 24 h after adding the antimetabolite (Figure 1(g)). Oligodendrocyte sensitivity to 6AN was further confirmed by sharp declines of the oligodendrocyte marker MBP that was observed in 6AN-treated differentiated OPCs. Conversely, no cytotoxicity was observed in primary cultures of Schwann cells that were exposed to 6AN for up to 3 days (Figure 1(h)). Such a reduced sensitivity suggests lesser role for NADPH in survival of Schwann cells as compared with OPCs or OPC-derived oligodendrocytes.

### Compromising NADPH Supply Induces Oxidative Stress

**Figure 2.**
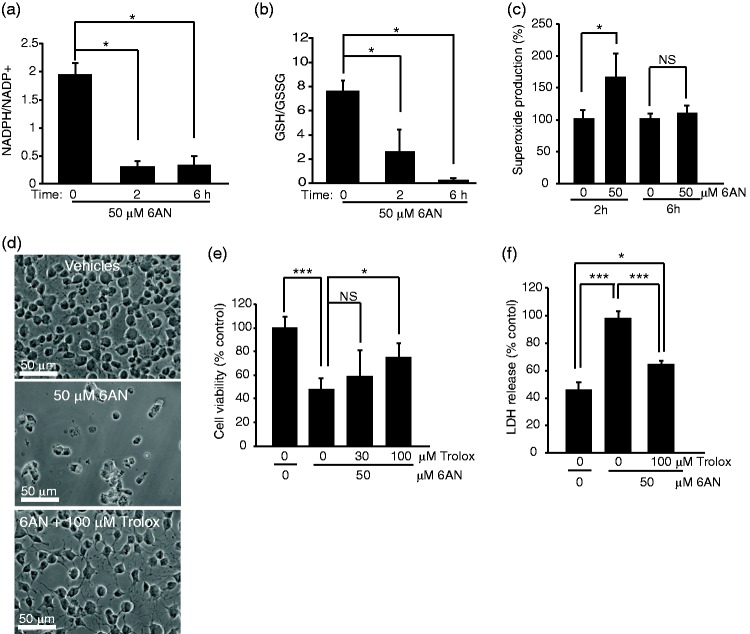
The NADPH antimetabolite 6-amino-NADP (6AN) compromises antioxidant defenses and induces oxidative cytotoxicity in oligodendrocyte precursor cells. Rat OPCs were treated as indicated; in (d–f), treatment times were 24 h (d–e) or 12 h (f). (a–b) Measurements of NADPH/NADP + and GSH/GSSG ratios. As early as 2 h after treatment with 50 μM 6AN, both ratios were dramatically reduced. (c) NBT assay revealed increased superoxide levels at 2 h after adding 6AN. Lack of effect at 6 h is likely due to damage of mitochondria which are the main source of superoxide in most cells (Figure 1(e)–(f)). Hence, compromised supply of antioxidant molecules NADPH and GSH resulted in oxidative stress preceding 6AN-induced cell death. (d–f) The antioxidant drug trolox reduced OPC toxicity of 6AN. (d) Representative phase contrast micrographs reveal that after a 24 h cotreatment the 6AN-induced decline in culture density was attenuated by 100 μM trolox. (e) In 6AN-treated OPCs, MTT survival assays showed protective effects of 100 but not 30 μM trolox. (f) LDH release assay indicated that 6AN-induced permeabilization of the plasma membrane was reduced by 100 μM Trolox. Data represent averages ± *SD* of three independent experiments (a, b) or nine sister cultures from three independent experiments (c), (e), (f); *ns, p* > .05; *p < .05; ***p < .001 (*U* test in (a), (b); analysis of variance in (c), (e), (f)).

To determine the cause of 6AN cytotoxicity, levels of antioxidant molecules NADPH and GSH were determined in 6AN-treated OPCs. The ratio of NADPH to NADP declined by 80% as early as 2 h after addition of 50 μM 6AN to the culture media (Figure 2(a)). Moreover, the ratio of reduced- to oxidized glutathione (GSH/GSSG) was reduced by 70% (Figure 2(b)). These observations suggest that in rat OPCs, NADPH supply is critical for restoration of GSH. As GSH is a key component of cellular antioxidant defenses, its depletion is expected to trigger oxidative stress. Indeed, increased production of a highly cytotoxic ROS, superoxide was observed in OPCs that were treated with 6AN for 2 h (Figure 2(c)). The increase reached 1.6-fold controls. After 6 h treatment with 6AN, superoxide levels normalized back to control levels (Figure 2(c)). As in most cells mitochondria are the main site of superoxide generation, such a reduction in superoxide content is likely a consequence of advanced mitochondrial damage. In support of this notion, 6AN treatment resulted in drop of mitochondrial potential at 6 h but not 2 h (Figure 1 and data not shown).

To determine whether 6AN-induced oxidative stress is cytotoxic, OPCs were treated with 6AN together with the ROS scavenger drug trolox for 24 h. At 100 μM, trolox reduced declines in OPC culture density as determined by phase contrast microscopy (Figure 2(d)). It also increased survival of 6AN-treated cells from 50% to 75% controls (Figure 2(e)). Finally, in OPCs that were treated with 6AN for 12 h, trolox attenuated plasma membrane permeabilization as indicated by 38% reduction of LDH release (Figure 2(f)). Therefore, in OPCs, the NADPH antimetabolite 6AN compromised antioxidant defenses and induced oxidative cytotoxicity.

**Figure 3.**
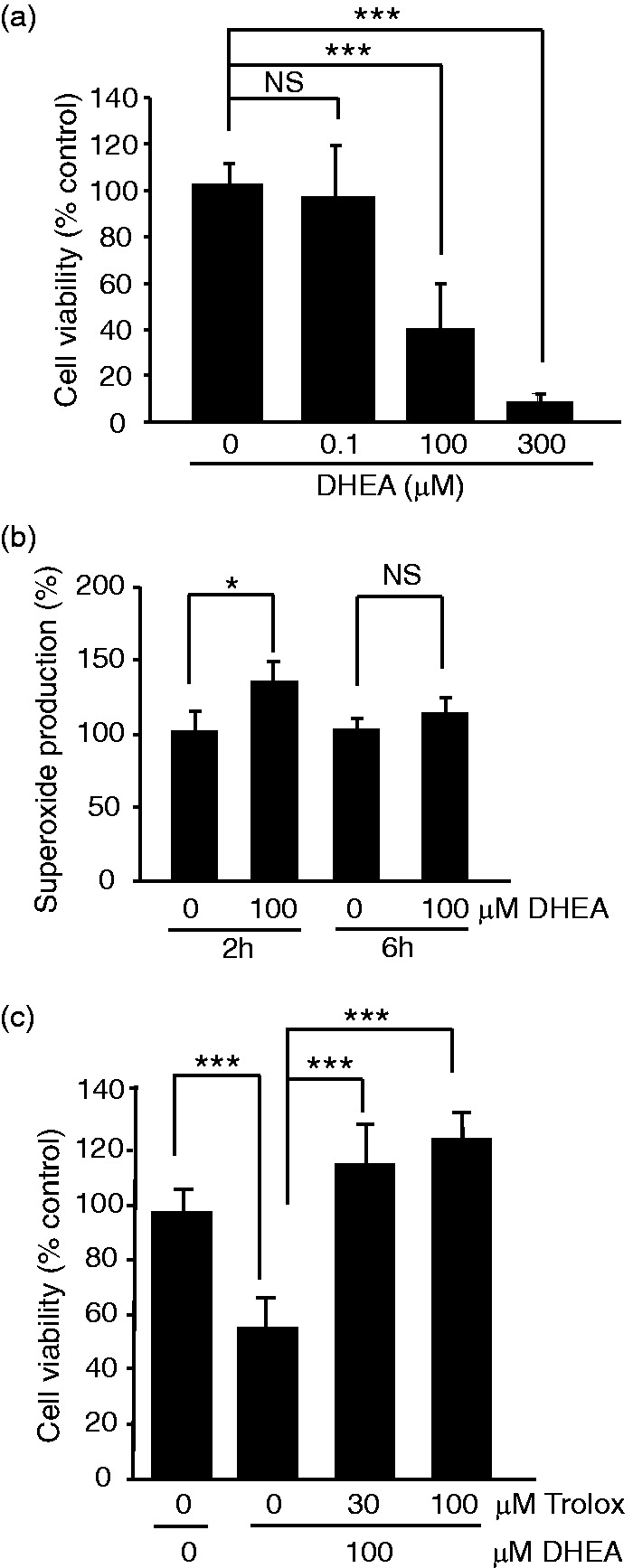
At G6PDH-inhibiting concentrations, dehydroepiandrosterone induces oxidative toxicity in oligodendrocyte precursor cells. OPCs were treated as indicated; treatment time in (a), and (c), was 24 h. (a) MTT cell survival assays revealed declining number of viable OPCs in response to 100 or 300 μM DHEA. (b) As in the case of 6AN, 2 h treatment with 100 μM DHEA increased superoxide levels. Normalized levels of superoxide at 6 h suggest mitochondrial dysfunction. (c) The antioxidant drug trolox protected against DHEA cytotoxicity. Data represent averages ± *SD* of nine sister cultures from three independent experiments; *ns, p* > .05, *p < .05, ***p < .001 (analysis of variance in (a), (c); *U* test in (b)).

To further confirm that 6AN effects are directly related to interference with NADPH, OPCs were treated with high concentrations of DHEA. At concentrations exceeding 10^−5^ M, DHEA is known to inhibit the oxidative arm of the PPP cycle compromising cellular NADPH supply. Indeed, similarto effects of 6AN, 24 h treatment with 100- or 300-μM DHEA reduced OPC survival to 40% or 5%, controls, respectively (Figure 3(a)). In addition, the 2 but not 6 h treatment with DHEA increased superoxide levels in OPCs (Figure 3(b)). Finally, the antioxidant trolox prevented DHEA-induced decline in OPC viability as revealed by MTT assay (Figure 3(c)). Taken together, NADPH supply appears to be critical for oligodendrocyte antioxidant defenses.

### Protection of NADPH-Depleted OPCs by Nanomolar Levels of DHEA

**Figure 4.**
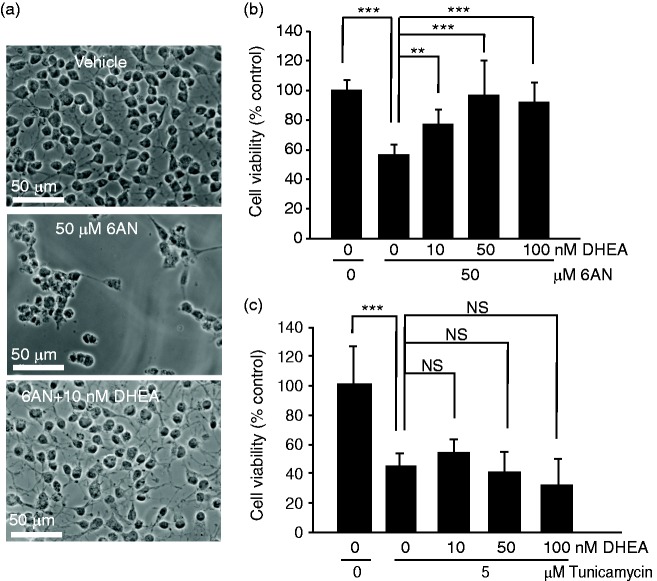
At physiologically relevant concentrations, dehydroepiandrosterone protects NADPH-depleted oligodendrocyte precursor cells. Cells were treated for 24 h as indicated. (a) Representative phase contrast micrographs of OPCs that were treated with low concentration of DHEA (10 nM) in combination with 50 μM 6AN. (b) MTT viability assays revealed that cotreatment with 6AN and low concentrations of DHEA reduced cytotoxicity of 6AN. (c) However, DHEA was ineffective against cytotoxicity of tunicamycin which kills OPCs by inducing ER stress. Data represent averages ± *SD* of nine sister cultures from three independent experiments, *ns, p* > .05, **p < .01; ***p < .001 (analysis of variance).

While DHEA may be used at very high concentrations to inhibit the PPP, it also promotes survival of various neural cells when applied in physiologically relevant concentration range of 10^−9^ to 10^−8^ M (Charalampopoulos et al., 2008; Maninger et al., 2009). Moreover, low doses of DHEA increased white matter sparing and improved locomotor outcome of experimental SCI. Therefore, we tested effects of 10^−8^ to 10^−7^ M DHEA in OPCs that were challenged with 6AN. At such concentrations, DHEA attenuated toxicity of 6AN (Figure 4(a) and (b)). For instance, 10 nM DHEA increased survival of 6AN-treated OPCs from 55% to 79% (Figure 4(b)). However, no prosurvival effects of DHEA were observed when OPCs were killed with the ER stress inducer tunicamycin (Figure 4(a) and (c)). Therefore, at nM concentrations, DHEA reduces the toxicity of NADPH depletion but not ER stress.

**Figure 5.**
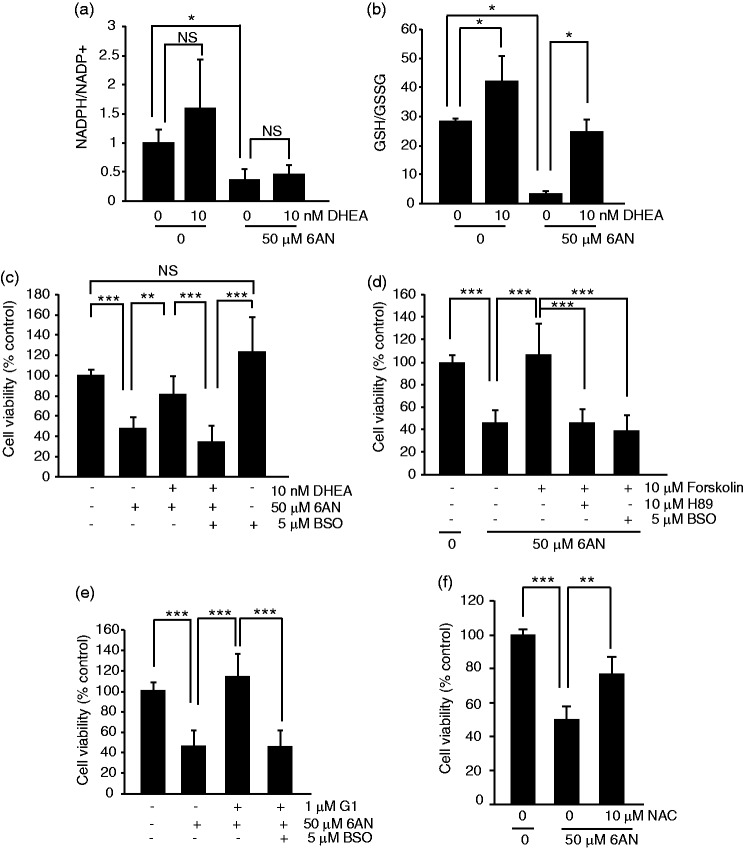
Requirement of glutathione synthesis for protection of NADPH-depleted oligodendrocyte precursor cells. OPCs were treated as indicated for 6- ((a–b) or 24 h (c–f)). (a) DHEA did not prevent decline of the NADPH/NADP^+^ ratio after PPP inhibition with 6AN. (b) DHEA increased the GSH/GSSG ratio under basal conditions and partially attenuated its decrease in response to 6AN. (c) MTT survival assay revealed that DHEA protection of 6AN-treated OPCs was abolished in the presence of the glutathione synthase inhibitor BSO. (d) Forskolin protected PPP-inhibited OPCs. The protection was sensitive to the PKA inhibitor H89 and the glutathione synthase inhibitor BSO. (e) G1, a selective agonist of GPER, protected 6AN-treated OPCs in a glutathione synthase-dependent manner; conversely, PKA was not involved in G1-mediated protection (data not shown). (f) Cotreatment with a GSH precursor, N-acetyl-L-cysteine (NAC) reduced 6AN toxicity. In (a–b), averages ±  *SD* of three independent experiments are shown; *ns, p* > .05; *p < .05 (*U* test); in (c–f), data represent averages ± *SD* of nine sister cultures from three independent experiments; *ns, p* > .05, **p < .01; ***p < .001 (analysis of variance).

A selective protection of cells that are challenged with oxidative but not ER stress suggests that 10^−8^ to 10^−7^ M DHEA may stimulate antioxidant defenses. At 10 nM, DHEA did not prevent 6AN-induced decline of NADPH/NADP ratio (Figure 5(a)). Instead, DHEA rescued 6AN-induced decrease of GSH/GSSG ratio suggesting increased supply of reduced glutathione (Figure 5(b)). It also increased GSH/GSSG ratio on its own (Figure 5(b)).

Increased levels of reduced glutathione despite depletion of NADPH suggest that DHEA may protect OPCs from 6AN-induced oxidative stress by either increasing *de-novo* synthesis of glutathione or reducing its usage. Therefore, the glutathione synthesis inhibitor BSO was used to test the first possibility. A 24-h treatment with BSO did not compromise OPC survival (Figure 5(c)). While DHEA rescued OPCs from 6AN toxicity raising survival from 48% to 82%, that effect was prevented by cotreatment with BSO (6AN + DHEA+BSO, 37% survival, Figure 5(c)). Therefore, *de-novo* synthesis of glutathione is required for DHEA-mediated protection of PPP inhibited OPCs.

Besides DHEA, injury-related white matter damage can be improved by stimulation of the cAMP pathway or the membrane estrogen receptor, G protein-coupled estrogen receptor (GPER/GPR30; Fiore et al., 2004; Pearse et al., 2004; Beaumont et al., 2009; Hu et al., 2012). Therefore, one could consider a possibility that these various pathways may converge on glutathione homeostasis to promote OPC or oligodendrocyte survival under oxidative stress due to NADPH depletion. Consistent with such a notion, the cAMP activator forskolin strongly protected 6AN-poisoned OPCs (Figure 5(d)). The protection was removed by BSO (Figure 5(d)). It was also sensitive to a protein kinase A (PKA) inhibitor, H89 (Figure 5(d)). Likewise, a specific GPER ligand, G1 protected OPCs from 6AN toxicity in a BSO-sensitive manner (Figure 5(e)). However, despite the fact the GPER is known to stimulate the cAMP-PKA pathway (Prossnitz and Barton, 2014), H89 did not affect the protection by G1 (data not shown).

**Figure 6.**
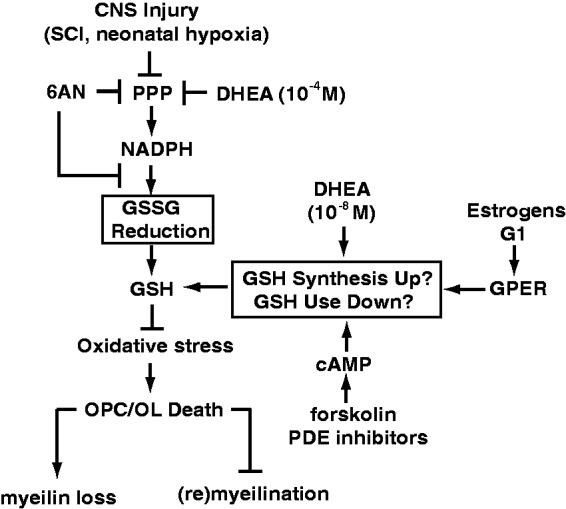
A hypothetical model proposing maintenance of glutathione homeostasis as a common effector for various interventions that support the survival of NADPH-depleted OPCs including physiological levels of DHEA, stimulation of GPER or activation of the cAMP pathway. Increased *de-novo* glutathione synthesis or its lower use may, at least temporarily, provide sufficient supply of reduced glutathione (GSH) despite disruption of the NDAPH-dependent reduction of oxidized glutathione (GSSG). In this way, the requirement of NADPH (and the PPP) for maintenance of redox homeostasis can be bypassed, and OPCs may survive the metabolic stress. We propose that such a protective mechanism may promote white matter sparing or repair following central nervous system injuries that are associated with oxidative stress-mediated death of OLs/OPCs. At least some of these insults may directly affect NADPH supply by lowering activity of PPP (see the Discussion section for more details).

If glutathione synthesis is critical for survival of NADPH-depleted OPCs, one could expect that providing a rate-limiting substrate(s) for this process will attenuate 6AN toxicity. Indeed, treatment with the amino acid N-acetyl-L-cysteine (NAC) which is a precursor for the key glutathione synthesis substrate L-cysteine reduced 6AN toxicity by at least 50% as determined using the MTT viability assay (Figure 5(f)). Thus, the presented findings suggest that while NADPH is required for antioxidant defenses of OPCs, such a requirement may be at least temporarily bypassed by increased supply of reduced glutathione (Figure 6). Moreover, this protective mechanism may be engaged by various interventions that improve outcome of experimental SCI including treatment with DHEA, stimulation of the cAMP pathway, or activation of the GPER.

## Discussion

The presented results demonstrate that after depletion of NADPH, both OPCs and oligodendrocytes undergo oxidative stress-mediated death. Hence, NADPH supply appears to be critical for oligodendrocyte survival at various stages of their development. Conversely, Schwann cells were relatively resistant to NADPH depletion. Therefore, NADPH plays a greater role in antioxidant defenses in myelinating cells of the central nervous system than the peripheral nervous system.

How relevant is chemical depletion of NADPH in cultured OPCs or oligodendrocytes to white matter damage after central nervous system injury? First, 6AN is known to induce extensive oligodendrocyte death in rodents suggesting that at least in these animals the oligodendrocyte lineage requires NADPH supply for its survival (Miyoshi et al., 1978; Cheeseman and Hothersall, 1988). Second, PPP activity and the related capacity to produce NADPH are altered in various pathologies affecting white matter.

For instance, increase in glycolysis and profound reduction of PPP activity is an early response to brain hypoxia in neonate rats (Brekke et al., 2014). Such a response has been proposed to contribute to oxidative stress and persistent white matter damage that follow perinatal hypoxia. Likewise, in cultured neurons, excitotoxicity is associated with increased glycolysis at expense of reduced PPP activity (Rodriguez-Rodriguez et al., 2012). Such a metabolic switch is sufficient to induce oxidative stress and neuronal death. As excitotoxicity is well documented in white matter loss after traumatic injuries or hypoxia/ischemia, it is tempting to speculate that a similar proglycolytic pathway may also contribute to OPC or oligodendrocyte death in response to these insults. In addition, our preliminary studies suggest a 30% reduction of G6PDH activity at 6 h after moderate thoracic spinal cord contusion in mice (Kilanczyk and Hetman, unpublished observations). In cultured OPCs, the oxidative toxicity of DHEA suggests that as in most other cell types oligodendrocytic G6PDH is the rate-limiting enzyme of the PPP. Therefore, trauma-associated decline of G6PDH activity is likely to compromise NADPH supply of spinal cord cells including OPCs.

In mature brain, increased activity of the oxidative PPP appears to be a common response to oxidative stress in traumatic injury, hypoxia, or neurodegenerative diseases (Domanska-Janik, 1988; Bartnik et al., 2005; Dunn et al., 2014; Jalloh et al., 2015). Interestingly, genetic enhancement of PPP activity and treatment with NADPH have been reported to reduce stroke-related brain damage in rodents and nonhuman primates (Li et al., 2014, 2016). Our findings provide the first direct evidence for functional significance of NADPH homeostasis in survival of OPCs or oligodendrocytes. In addition, our observations support a notion that NADPH supply and the PPP may be adequate targets for therapies to reduce white matter damage after injury.

At physiologically relevant concentrations, the neurosteroid DHEA protected NADPH-depleted OPCs. To the best of our knowledge, this is the first direct demonstration of DHEA-mediated protection of glial precursor cells from a metabolic stress. However, it is well established that at such low concentrations that are close to physiological brain levels in rodents (0.1–1 nM) and humans (10–20 nM), DHEA supports survival of neurons as well as neural cell lines (Charalampopoulos et al., 2008; Maninger et al., 2009). For instance, DHEA protects against NMDA excitotoxicity (Kimonides et al., 1998). It also reduces cytotoxicity of hydrogen peroxide (Leskiewicz et al., 2008). Moreover, *in vivo* neuroprotection has been reported in stroke models (Aragno et al., 2000; Li et al., 2009). Finally, in the spinal cord, DHEA sulfate protected against ischemic damage while DHEA reduced white matter loss and improved functional recovery in a mouse contusive SCI model (Lapchak et al., 2000; Fiore et al., 2004; Compagnone, 2008). Importantly, in all these examples of cytoprotection, oxidative stress is a central component of cell or tissue injury. Therefore, DHEA may be selectively protective in such conditions that involve excessive oxidative stress.

DHEA is produced in the nervous system (Maninger et al., 2009). However, its levels decline with age. During development, DHEA seems to cooperate with the key motoneuron inducer Sonic Hedgehog (Shh) to promote neural precursor proliferation, differentiation, and morphogenesis (Compagnone and Mellon, 1998; Galdo et al., 2012). Our observations identify DHEA as an important antioxidant signal that protects adult spinal cord OPCs. These cells have an enormous potential of neurorepair that is mobilized in response to various injuries including ischemia (He et al., 2013). However, similarly to DHEA levels, remyelination potential decreases with age (Chari et al., 2003). Therefore, it is tempting to speculate that DHEA contributes to the age-restricted potential for remyelination by supporting OPC survival during injury.

What is the receptor that mediates prosurvival effects of DHEA in PPP-inhibited OPCs? One possibility is that DHEA is metabolized to estradiol or testosterone and acts on their respective receptors that are expressed in OPCs (Traish et al., 2011). Indeed, estradiol modulates OPC or oligodendrocyte survival, myelin loss, remyelination potential, or functional recovery following injuries that target the white matter (Morales et al., 2006; Gerstner et al., 2007, 2009; Acs et al., 2009; Mosquera et al., 2014). In some cases, including autoimmune encephalitis and contusive SCI, the beneficial effects of estradiol were mediated by estrogen receptor alpha (Morales et al., 2006; Mosquera et al., 2014). In addition, GPER supports OPC proliferation and accelerates remyelination in the cuprizone-induced demyelination model (Hirahara et al., 2013). DHEA and DHEA-BSA activate GPER in human hepatocellular carcinoma cell lines and primary human hepatocytes (Teng et al., 2015). Moreover, selective activation of GPER suppressed apoptosis and improved functional recovery after contusive SCI (Hu et al., 2012). However, in a SCI model, a DHEA analog that cannot be metabolized toward estrogen or testosterone had similar protective effects as DHEA (Compagnone, 2008). Therefore, DHEA-mediated improvement in SCI outcome may be mediated by its own receptors or by modulation of other ligand or receptor systems such as sigma, NMDA, GABA-A, and receptor tyrosine kinases (Charalampopoulos et al., 2008; Maninger et al., 2009). Future studies are needed to determine which of these receptors are involved in prosurvival effects of DHEA in OPCs.

OPC protection against PPP inhibition was also achieved by the adenylyl cyclase activator forskolin. Of note, activators of cAMP signaling have well-established positive effects on functional outcome of moderate experimental SCI (Pearse et al., 2004; Beaumont et al., 2009). Moreover, such effects of cAMP have been shown to be associated with increased survival of oligodendrocytes and white matter sparing (Pearse et al., 2004; Whitaker et al., 2008; Beaumont et al., 2009). Although, PKA, a principal transducer of many cAMP effects, was involved in forskolin-mediated protection, it appeared dispensable for protective effects of DHEA or G1 (data not shown). Conversely, forskolin, G1, and DHEA engaged a similar effector mechanism to protect NADPH-depleted OPCs. That effector mechanism was dependent on *de-novo* synthesis of glutathione as indicated by attenuated protection after glutathione synthesis inhibition or protective sufficiency of supplying the glutathione precursor NAC.

Taken together, our data illustrate the importance of NADPH supply for antioxidant defenses and survival of adult spinal cord OPCs. Consequently, injury-associated inhibition of the PPP may be detrimental for the remyelination potential of the white matter. However, steroid hormones and cAMP activators may help to preserve such a repair capacity by increasing a NADPH-independent supply of reduced glutathione. Therefore, glutathione homeostasis appears as a critical effector mechanism for OPC protection against NADPH depletion. Finally, interventions that promote glutathione homeostasis may be a key component of combinatorial therapies to improve outcome in such white matter-destroying injuries as spinal cord trauma, stroke, or perinatal hypoxia.
